# Dietary Protein and Fat Intake Affects Diabetes Risk with *CDKAL1* Genetic Variants in Korean Adults

**DOI:** 10.3390/ijms21165607

**Published:** 2020-08-05

**Authors:** Woo Jeong Choi, Hyun-Seok Jin, Sung-Soo Kim, Dayeon Shin

**Affiliations:** 1 Department of Food and Nutrition, Inha University, 100 Inha-ro, Michuhol-gu, Incheon 22212, Korea; yyb01019@naver.com; 2 Department of Biomedical Laboratory Science, College of Life and Health Sciences, Hoseo University, Asan, Chungnam 31499, Korea; jinhs@hoseo.edu (H.-S.J.); kims7152@naver.com (S.-S.K.)

**Keywords:** *CDKAL1* variants, single nucleotide polymorphisms, diabetes, Korean Genome and Epidemiology Study (KoGES)

## Abstract

Cyclin-dependent kinase 5 regulatory subunit-associated protein 1-like 1 (*CDKAL1*) is one of the strongest diabetes loci identified to date; evidence suggests that it plays an important role in insulin secretion. Dietary factors that affect insulin demand might enhance the risk of diabetes associated with *CDKAL1* variants. Our aim was to examine the interactions between dietary protein and fat intake and *CDKAL1* genetic variants in relation to the risk of diabetes in Korean adults. Single nucleotide polymorphisms (SNPs) were selected with a genome-wide association study (GWAS) for diabetes after adjustment for age, gender, and examination site. Using data from the Health Examinees (HEXA) Study of the Korean Genome and Epidemiology Study (KoGES), 3988 middle-aged Korean adults between 40–76 years of age (2034 men and 1954 women) were included in the study. Finally, rs7756992 located within the *CDKAL1* gene region was selected from GWAS (*p*-value < 5 × 10^−8^). Multivariable logistic regression models were used to evaluate the interactions between genotypes and dietary protein and fat intake in relation to diabetes risk after adjustment for age, gender, BMI, waist circumference, physical activity, smoking status, drinking habits, and examination site. Significant interactions between *CDKAL1* rs7756992 and dietary protein and fat intake for the risk of diabetes were observed in men (*p*-value < 0.05). In women, significant interactions between dietary protein and fat intake and *CDKAL1* variants (rs7756992) were associated with increased risk of diabetes (*p*-value < 0.05). Dietary protein and fat intake interacted differently with *CDKAL1* variants in relation to the risk of diabetes in Korean adults of both genders. These findings indicate that *CDKAL1* variants play a significant role in diabetes and that dietary protein and fat intake could affect these associations.

## 1. Introduction

Diabetes is a rapidly growing public health issue which is associated with morbidity and premature mortality [1]. Prevalence of diabetes is greater among Asians than in other populations from the rest of the world [2]. According to the Korea Health Statistics 2018, prevalence of diabetes in individuals aged 30 years and older (age standardized) was 12.4% (based on fasting blood glucose) and 13.8% (based on fasting blood glucose and glycated hemoglobin). Thus, more than one in 10 adults aged 30 years and older are diagnosed with diabetes mellitus in Korea [3].

Most individuals with type 2 diabetes suffer from serious complications such as nephropathy, neuropathy, retinopathy, cardiovascular disease, and dysfunction of pancreas, skeletal muscle, and liver physiology [4,5]. Diabetes is a complex disease caused by interactions between multiple genetic and environmental risk factors [6]. People with a family history of diabetes show genetic propensity to develop diabetes than those who do not [7,8]. It is also established that various factors such as dietary intake, physical fitness, and emotional states can act as risk factors [9,10]. Genetic susceptibility synergistically acts in addition to environmental factors, leading to diabetes development [11].

Recently, many novel susceptibility genes for diabetes have been identified by meta-analyses of genome-wide association studies (GWAS) [12]. Among these, the cyclin-dependent kinase 5 regulatory subunit-associated protein 1-like 1 (*CDKAL1*) gene spans 697,948 bp on chromosome 6p22.3 and encodes a 65 kDa protein. The *CDKAL1* gene plays a role in suppressing complex of CDK5-p35 related beta cells function [13]. *CDKAL1* variants are associated with beta-cell disorder phenotypes, in particular altered insulin release, detected by hyperglycemic clamp or glucose tolerance test [14,15,16]. The carriers of *CKDAL1* variants associated with diabetes are related to decreased HOMA-*β* implying beta cell dysfunction [17]. SNP rs7756992 A/G polymorphism is in intron of the *CDKAL1* gene. Previous research suggests that *CDKAL1* rs7756992 is associated with type 2 diabetes in several populations [17,18].

Prevalence of single risk factors and clinical aspects in Korea may be different from that in western countries. The interaction between genetic disposition and environmental factors, in particular, dietary habits, plays a significant role in diabetes development [19,20]. Variants of the gene encoding the brain-derived neurotrophic factor (*BDNF*) were found to show significant associations with energy and protein intake in Korean adults with type 2 diabetes [21]; furthermore, a study on the Canadian population examined the reactivity of transcription factor 7-like 2 (*TCF7L2*) to dietary fat intake, influencing insulin sensitivity and glucose tolerance [22]. However, only limited studies have evaluated the interactions between dietary protein and fat intake and *CDKAL1* genetic variations in relation to the risk of diabetes. In animal study, glucose tolerance and insulin secretion were damaged by defection of *CDKAL1* in mice, and glucose tolerance and insulin sensitivity were more degenerated in mice with higher dietary fat intake during twenty weeks [23]. The aim of this study was to explore the interactions between dietary protein and fat intake and *CDKAL1* genetic variants in the Korean adult population, to better understand their role in diabetes development.

## 2. Results

### 2.1. General Characteristics of the Study Population in Cases and Controls

We investigated 3988 participants of the Health Examinees (HEXA) Study of the Korean Genome and Epidemiology Study (KoGES), which included 2034 men (1297 with diabetes, 737 without diabetes) and 1954 women (1207 with diabetes, 747 without diabetes). In men, smoking status, physical activity, and drinking habits did not differ between cases and controls. HDL-cholesterol level was lower in cases (46.4 mg/dL) than in controls (51.6 mg/dL) (*p*-value < 0.05). Triglyceride (TG) level, BMI, and waist circumference were significantly higher in cases than in controls (*p*-value < 0.05) (Table 1). Smoking, physical activity, and drinking habits did not differ between female cases and controls. Similarly, in men, women had a higher HDL-cholesterol level in the control group than in the case group, while the TG level was significantly higher in cases than in controls (*p*-value < 0.05). BMI and waist circumference of women were also significantly higher in cases than in controls (*p*-value < 0.05) (Table 1). In total population, cases had higher TG level and lower HDL-cholesterol level than controls (*p*-value < 0.05).

### 2.2. Association of SNPs in CDKAL1 Gene with Fasting Blood Glucose and Glycated Hemoglobin

Among the 83 SNPs, only seven reached the significance threshold *p*-value (5 × 10^−8^) and were analyzed for association with fasting blood glucose and glycated hemoglobin (HbA1c) levels. We used linear regression analysis after adjusting for gender and age. The genetic model was based on an additive genetic model. Thus, seven SNPs (rs7756992, rs9368222, rs2206734, rs9465871, rs7747752, rs9356744, and rs6908425) were significantly associated with the risk of diabetes and fasting blood glucose. A total of six SNPs (rs7756992, rs9368222, rs2206734, rs9465871, rs7747752, and rs9356744) showed significant association with fasting blood glucose and HbA1c (all *p*-values < 0.05); only rs6908425 was not significantly associated with HbA1c (*p* = 0.227) (Table 2).

Supplementary Table S1 compares the results of the logistic regression analyses between *CDKAL1* SNPs and diabetes after applying either a co-dominant, dominant, or recessive model. Each SNP was significant for diabetes in all three models (*p* < 0.05). Supplementary Table S2 shows linear regression analysis between SNPs in *CDKAL1* and fasting blood glucose and glycated hemoglobin, and all associations were significant (*p* < 0.05).

### 2.3. Associations between CDKAL1 SNPs and Diabetes in Korean Population

**Figure 1:**
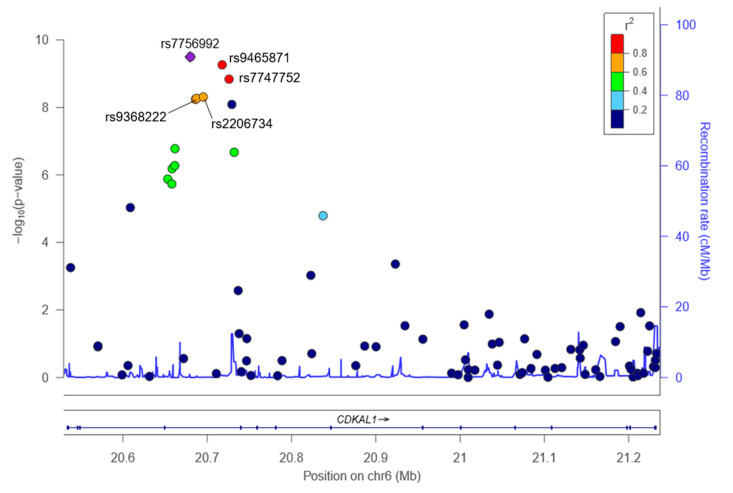
Regional plot for *CDKAL1*_rs7756992, rs9465871, rs7747752, rs2206734, and rs9368222. The positions of the SNPs are shown at the top of the figure, and associations between SNPs in the CDKAL1 gene and diabetes are shown in the middle. The statistical significances (−log_10_ *p*-value) of associations with the SNPs are plotted. The recombination rates estimated using 1000 Genomes Nov 2014 Asian population data are shown by a blue line. The purple diamond with a SNP number represents the SNP most strongly associated with diabetes, and its correlations with other SNPs are shown by colors indicating the levels of linkage disequilibrium (*r*^2^). SNP map on chromosome 6p20: polymorphisms identified.

We confirmed the relationship between five SNPs with a regional plot, set a *CDKAL1* gene as the standard, and set the flanking size to 5 kb (Figure 1). Based on rs7756992, which had the lowest *p-*value (3.16 × 10^−10^), the level of linkage disequilibrium (*r*^2^) for rs9465871 and rs7747752 was high (>0.8), and three SNPs (rs7756992, rs9465871 and rs7747752) were associated with decreased risks of diabetes (*p* = 3.16 × 10^−10^, 5.46 × 10^−10^, and 1.45 × 10^−9^); for possession of minor allele, OR 0.73, 0.73, and 0.74. While based on rs7756992, *r*^2^ of rs2206734 and rs9368222 was >0.6, and two SNPs (rs2206734, rs9368222) were associated with increased risks of diabetes (*p* = 4.91 × 10^−9^ and 5.40 × 10^−9^); for possession of minor allele, OR 1.35 and 1.35 (Table 2).

### 2.4. Interactions of SNP and Protein Intake (% Energy) in Relation to the Risk of Diabetes

Men in the first tertile of protein intake (% energy) after multivariable adjustment (age, examination site, BMI, smoking status, drinking habits, and physical activity) showed diabetes association with rs7756992 (*p* for trend = 0.034); the SNP had significantly higher adjusted odds ratio (AOR) when possessing a risk allele (AOR 2.04, 95% confidence intervals [CI] 1.16–3.60). Men in the second tertile group having the rs7756992 GG allele had higher AOR 1.91 (95% CI 1.02–3.58) compared to those with the AA allele (Table 3).

Women in the third tertile of protein intake (% energy) after multivariate adjustment showed diabetes association with rs7756992 (*p* for trend = 0.011). Women in the first tertile group with the rs7756992 GG allele had higher AOR 1.88 (95% CI: 1.01−3.50) compared to those with the AA allele. In the third tertile group with the rs7756992, AG allele and GG allele had higher AORs, 2.08 (95% CI: 1.12–3.88) and 2.29 (95% CI: 1.16–4.53), compared to those with AA allele (Table 3). 

The total in the first tertile of protein intake (% energy) after multivariate adjustment showed diabetes association with rs7756992 (*p* for trend = 0.001). The total in the first tertile group with the rs7756992 GG allele had higher AOR 2.05 (95% CI: 1.35–3.09) compared to those with the AA allele (Table 3).

### 2.5. Interactions of SNP and Fat Intake (% Energy) in Relation to the Risk of Diabetes

Men in the first tertile of fat intake (% energy) after multivariate adjustment showed diabetes association with rs7756992 (*p* for trend = 0.009). Men in the first tertile group with the GG allele had higher AOR 2.33 (95% CI: 1.34–4.05) compared to those with AA allele (Table 4).

Women in the second tertile of fat intake (% energy) after multivariate adjustment did not show diabetes association with rs7756992 (*p* for trend = 0.089). Women with the rs7756992 GG allele in the second and third tertiles did not have significant association with diabetes risk; however, they had high AORs, 1.86 (95% CI: 1.00–3.44) and 2.26 (95% CI: 1.11–4.59), compared to those with the AA allele (Table 4).

Total in the first and second tertiles of fat intake (% energy) after multivariate adjustment showed diabetes association with rs7756992 (*p* for trend = 0.009 and 0.044, respectively). Total with the rs7756992 GG allele in the first, second and third tertiles had higher AORs, 1.63 (95% CI: 1.08–2.45), 1.58 (95% CI: 1.03–2.41), and 1.67 (95% CI: 1.05–2.66), compared to those with the AA allele (Table 4).

## 3. Discussion

Significant relationships between *CDKAL1* genetic variants (rs7756992) and dietary protein and fat intake in relation to diabetes were observed in the Korean population from the KoGES-HEXA study. Diabetes is caused by genetic and environmental factors, and we found a significant interaction between dietary protein and fat intake and *CDKAL1* genetic variants in relation to the risk of diabetes. In men, significant associations between rs7756992 and lower dietary protein and fat intake were observed. In women, significant associations between rs7756992 and higher dietary protein and fat intake were observed.

In the present study, women with risk alleles in the third tertile of protein intake were increasingly at risk for diabetes. The interaction between high protein intake and diabetes risk has been well documented in previous studies [24,25,26,27,28]. In a study on Europeans, high total dietary protein (protein substitution for fat and carbohydrates) increased diabetes development rates [24]. In addition, the incidence of type 2 diabetes was higher in those with high total protein intake (hazard ratio [HR] 1.06 [95% CI 1.02–1.09], *p* for trend < 0.001). Based on another study on European populations, a higher total protein intake is associated with type 2 diabetes development; all associations were stronger in women with obesity [25]. In men and women from the US, groups with higher total protein intakes were associated with increased diabetes risk [26]. Consistently with this finding, high protein intake was related to increased risk of diabetes in South Asian Indians (OR: 1.47–1.85, 95% CI: 1.02–2.84) [27]. Linn et al. [28] reported that high protein diets increase glucose-stimulated insulin release (*p* = 0.012) because of decreased glucose limit point of the incretion *β*–cells (*p* = 0.031) [28]. 

Our findings indicate that women in the third tertile fat intake with risk alleles had increased diabetes risk. Previous findings on diabetes risk related to increased fat intake are also well documented. In a multinational study from 2003, including six countries (Greece, Italy, Algeria, Bulgaria, Egypt, and Yugoslavia), animal fat intake was associated with increased incidence of diabetes [29]. In US populations, total fat intake was significantly related to increased risk of diabetes (AOR 1.27, 95% CI 1.04–1.55, *p* for trend = 0.02) [30]. In particular, high meat consumption and a high saturated fat diet both predisposed to diabetes [30]. Parallel with previous findings, fat ingestion was significantly associated with type 2 diabetes mellitus risk in subjects from the rural populations of San Luis Valley in Colorado with disturbed glucose tolerance [31]. Fasting and postprandial glucose, and glycated hemoglobin levels were studied corresponding to different proportions of dietary carbohydrate and fat intake in Korean patients with non-insulin-dependent diabetes mellitus [32]. The studies showed that those with the lower carbohydrate intake and the higher fat intake had the lower fasting glucose, postprandial glucose and glycated hemoglobin levels in both men and women [32]. A previous study has also shown that maintaining a dietary pattern based on less carbohydrates and more fat may reduce the risk of diabetes in the US population [33]. 

*CDKAL1* is a strong gene identified so far as being significantly associated with diabetes. *CDKAL1* variants have been shown to predict the development of diabetes in individuals with impaired insulin secretion, which suggests potential synergistic effects between different risk factors [16,23]. SNP rs7756992 observed in the present study was associated with diabetes-associated indicators. A significant association was observed between the rs7756992 *CDKAL1* gene variant and the risk of diabetes. Similar to our finding, individuals with rs7756992 G allele polymorphism were susceptible to diabetes [34,35]. In the Russian population, those who retained G/G allele of rs7756992 had higher OR 1.59 (95% CI 1.10–2.29, *p*-value < 0.05) compared to those who retained A/A allele [17]. Steinthorsdottir et al. [16] reported that homozygous carriers of the risk allele G of rs7756992 had a 22% decreased insulin response to glucose load than A/A allele carriers. Previous findings suggest significant associations between *CDKAL1*, alcohol intake, dietary fat, and energy intake in relation to diabetes [23,36,37]. In the Ansan/Ansung Korean cohort, diabetes risk increased by 1.549 (95% CI 1.207–1.720) when high alcohol intake was combined with the presence of *CDKAL1* risk alleles [36]. In Japanese men, the interaction between *CDKAL1* gene variants and excessive energy intake increased glycated hemoglobin (*p* = 0.037) [37].

The mechanism on how *CDKAL1* interacts with dietary fat and protein intake for type 2 diabetes remains unclear. Dietary factors play a role in the relationship between *CDKAL1* polymorphism and diabetes. *CDKAL1* polymorphisms may modulate insulin resistance in response to the different levels of dietary fat and protein intake. For example, previous research found that perilipin (*PLIN*) genetic variants (11482GA and 14995AT) modulated the effects of dietary fat and carbohydrate consumption on insulin resistance in a large sample of Asian female population, indicating a significant gene–diet interaction [38].

To the best of our knowledge, this is the first study to examine *CDKAL1*-dietary interactions using data from KoGES-HEXA. The present study has several strengths such as the large sample size and inclusion of several potential covariates that affect the relationships between dietary factors, genetic variants, and diabetes. However, several limitations need to be considered when interpreting the results. First, as we conducted cross-sectional analyses using baseline data from KoGES-HEXA, any conclusion about strict cause–effect relationships between dietary factors, genetic variants, and diabetes risk cannot be drawn. Second, we set the age condition of controls (no diabetes) to be 60 years old or older. According to a study, acquired diabetes patients account for more than 50% diabetes patients over 60 years of age [39]. In our study, the age of controls was set at 60 or older, assuming those without diabetes in their 60s and older are more likely to have a genetic background that would not predispose to diabetes. Third, there are only two clinical criteria for determining diabetes (fasting blood glucose and glycated hemoglobin). However, in cohort documents, two more useful clinical indicators were employed to diagnose diabetes. Moreover, according to the Korea Diabetes Association, fasting blood glucose and glycated hemoglobin tests are primarily conducted during diabetes screening [40], so we conducted the study by combining both diabetes history and the two clinical indicators together.

## 4. Materials and Methods

### 4.1. Study Population

The data used in this study were collected from the HEXA cohort of the KoGES from 2004 to 2016 (Seoul, Busan, Daegu, Gwangju, Ulsan, Anyang, Gyeonggi province, Chuncheon, Gangwon province, Cheonan, Chungnam province, Masan, and Gyeongnam province). KoGES-HEXA targeted individuals over 40 years old, living in urban cities; all subjects were examined at medical centers to construct the epidemiological infrastructure necessary to correlate environmental and genetic factors with common chronic diseases in urban cities.

**Figure 2:**
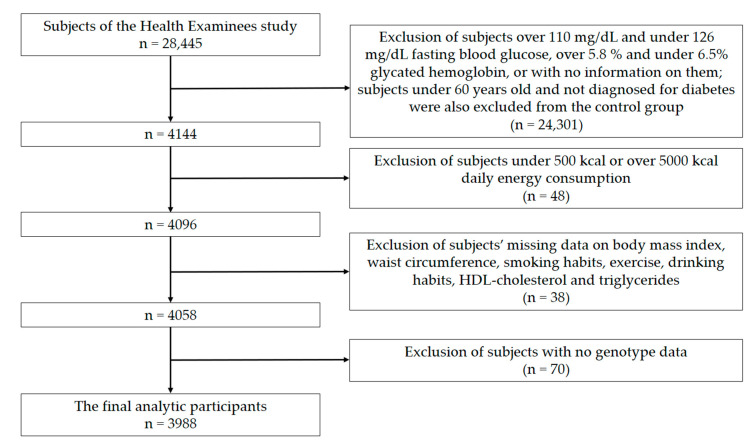
Flowchart of the study population.

This study used KoGES-HEXA data from years 2004–2013 (n = 28,445). Among these HEXA cohort populations, 24,301 people with no data on diabetes were excluded from both cases and/or controls; subjects under 60 years old were excluded from the control group. Forty-eight people who had inadequate energy intake (< 500 kcal or > 5000 kcal), 38 people with missing data (about drinking habits, smoking status, physical activity, anthropometric measurements, and biochemical variables), and 70 people missing genotype data were excluded. Thus, 3988 individuals (Figure 2) constituted the actual analytic study group. The study protocol was reviewed and approved by the Institutional Review Board (IRB) of Inha University on January 31, 2020 (IRB No. 200129–1A). 

### 4.2. General Characteristics, Anthropometric Measurements, and Biochemical Variables

We surveyed general information of subjects, including age, gender, smoking status (current, past, none), drinking habits (current, past, none), and physical activity (yes, no); anthropometric measurements, including waist circumference, height, weight; and biochemical variables (HDL-cholesterol and triglycerides). Body mass index (BMI, kg/m^2^) was calculated on the basis of the height (m) and weight (kg) of study participants.

### 4.3. Diabetes Detection

Patients with diabetes were defined based on a previous diabetes diagnosis. Moreover, individuals who had fasting blood glucose ≥ 126 mg/dL or glycated hemoglobin (HbA1c) ≥ 6.5% were included as per the 2015 treatment guidelines for diabetes, from the Korean Diabetes Association (KDA) [40]. The non-diabetes group (controls) consisted of people with no diabetes diagnosis, fasting blood glucose < 110 mg/dL, glycated hemoglobin < 5.8%, and an age over 60.

### 4.4. Dietary Measurements

Dietary intake was assessed by using a food frequency questionnaire (FFQ), which included data about daily energy intake (kcal/day), daily protein intake (g/day), and daily fat intake (g/day). The percentages (%) of energy intake from protein and fat intake were calculated as follows: 1 g of protein and 1 g of fat was multiplied by 4 kcal and 9 kcal, respectively, to obtain the percentage of energy consumption.

### 4.5. Genotyping

A total of 28,445 samples were genotyped according to the manufacturer’s protocol, which recommended the Axiom^®^ 2.0 Reagent Kit (Affymetrix Axiom^®^ 2.0 Assay User Guide; Affymetrix, Santa Clara, CA, USA), and the genotype data were produced using the Korean-Chip, which is available through the Korean-Chip consortium. The Korean-Chip was designed by the Center for Genome Science at the Korea National Institute of Health (4845-301, 3000-3001). The detailed procedure was described in a previous report [41]. Samples that revealed the following features we excluded during the quality control process: sex inconsistency, markers with a high missing rate (>5%), individuals with a high missing rate (>10%), minor allele frequency <0.01, and a significant deviation from Hardy–Weinberg equilibrium (HWE) (*p* < 0.001).

After genotyping and sample quality control, GWAS was performed to select SNPs significantly associated with diabetes in KoGES-HEXA subjects after normalization for age, gender, and examination site (Bonferroni *p*-value < 5 × 10^−8^). Finally, rs7756992 located within the *CDKAL1* gene region was selected.

### 4.6. Statistical Analyses

PLINK (version 1.90 beta) was used for GWAS, in order to select SNPs associated with diabetes. Association of genetic variants with diabetic or control individuals was based on an additive genetic model and analyzed by logistic regression. Association of *CDKAL1* SNPs with fasting blood glucose and glycated hemoglobin levels was analyzed with a linear regression model after adjusting for age, gender and examination site.

We calculated frequency and percentage for each data category from the subjects (gender, smoking status, drinking habits, and physical activity) and conducted a chi-squared test to detect the significant associations between these categorical variables. We calculated mean and standard deviation for all other continuous variables (age, HDL-cholesterol, TG, BMI, waist circumference, daily energy intake, daily protein intake, and daily fat intake) and conducted *t*-tests to detect differences between cases and controls. Multivariable logistic regression models were evaluated for interactions between *CDKAL1* genetic variants and dietary protein and fat intake (% energy/day) in relation to the risk of diabetes after adjusting for age, BMI, examination site, smoking status, drinking habit, and physical activity. Intake (% energy/day) of dietary protein and fat was divided into tertiles. Statistical analyses were performed by using the PLINK and SPSS (Statistical Package for the Social Sciences) software (version 25.0; SPSS Inc., IBM, New York, NY, USA). Statistical significance was determined with two-sided *p*-value < 0.05. Web-based program Locuszoom version 1.3 (http://csg.sph.umich.edu/locuszoom/) was used to observe regional association plots.

## 5. Conclusions

In conclusion, dietary protein and fat intake interacted with *CDKAL1* variants in relation to the risk of diabetes, which does vary depending on gender. Patients with diabetes should undertake dietary control despite being treated with medication to manage their blood glucose. The current findings support the role of dietary protein and fat intake as useful indicators for diabetes risk in Korean men and women who have *CDKAL1* risk alleles. These findings would help public health professionals to detect high-risk individuals for diabetes with different responses to diet, and this can contribute to the development of more genetic-targeted dietary guideline for specific subpopulations. *CDKAL1* variants play a significant role in diabetes, and dietary protein and fat intake could impact their function. Recently, human islet 3D genome maps have been developed and validated to identify target genes for diabetes-relevant regulatory elements [42]. For future studies, polygenic risk score based on the combined set of risk variants through islet hub variants could be used to provide more insights on illuminating the pathophysiology on the genetics of diabetes.

## Figures and Tables

**Table 1:** General characteristics of study participants according to the absence and presence of diabetes in Korean men and women.

	Men (*n* = 2034)					Women (*n* = 1954)					Total (*n* = 3988)				
	No Diabetes (*n* = 737)		Diabetes (*n* = 1297)		*p-*Value ^1^	No Diabetes (*n* = 747)		Diabetes (*n* = 1207)		*p-* Value ^1^	No Diabetes (*n* = 1484)		Diabetes (*n* = 2504)		*p-*Value ^1^
Age (years)	64.1 ± 3.0		58.1 ± 7.4		<0.001	63.6 ± 2.9		57.8 ± 6.9		<0.001	63.9 ± 2.9		58.0 ± 7.1		<0.001
Smoking Status					<0.001					0.046					<0.001
Current	113	15.3%	340	26.2%		7	0.9%	30	2.5%		120	8.1%	370	14.8%	
Past	390	52.9%	609	47.0%		6	0.8%	12	1.0%		396	26.7%	621	24.8%	
None	234	31.8%	348	26.8%		734	98.3%	1165	96.5%		968	65.2%	1513	60.4%	
Physical Activity					0.278					0.180					0.747
No	250	33.9%	471	36.3%		345	46.2%	520	43.1%		595	40.1%	991	39.6%	
Yes	487	66.1%	826	63.7%		402	53.8%	687	56.9%		889	59.9%	1513	60.4%	
Alcohol Use					0.49					0.091					0.822
Current	504	68.4%	903	69.6%		151	20.2%	227	18.8%		655	44.1%	1130	45.1%	
Past	76	10.3%	113	8.7%		8	1.1%	29	2.4%		84	5.7%	142	5.7%	
None	157	21.3%	281	21.7%		588	78.7%	951	78.8%		745	50.2%	1232	49.2%	
HDL-Cholesterol (mg/dL)	51.6 ± 12.4		46.4 ± 11.0		< 0.001	55.4 ± 13.3		50.0 ± 11.4		<0.001	53.5 ± 13.0		48.1 ± 11.4		< 0.001
Triglyceride (mg/dL)	128.5 ± 81.3		171.3 ± 135.3		< 0.001	120.4 ± 69.3		151.3 ± 101.5		<0.001	124.5 ± 75.6		161.6 ± 120.6		< 0.001
BMI (kg/m^2^)	23.8 ± 2.6		25.1 ± 2.9		< 0.001	23.8 ± 2.7		25.2 ± 3.3		<0.001	23.8 ± 2.7		25.2 ± 3.1		< 0.001
Waist Circumference (cm)	84.7 ± 7.4		88.3 ± 7.7		< 0.001	79.6 ± 7.5		83.4 ± 8.4		<0.001	82.1 ± 7.9		85.9 ± 8.4		< 0.001
Total Energy Intake (kcal)	1749.8 ± 473.2		1795.5 ± 473.8		0.037	1585.9 ± 474.6		1629.6 ± 489.6		0.053	1667.3 ± 480.8		1715.5 ± 488.5		0.002
Total Carbohydrate Intake (gram)	314.9 ± 79.3		318.3 ± 77.9		0.351	289.2 ± 84.1		297.6 ± 84.6		0.033	302.0 ± 82.7		308.3 ± 81.8		0.019
Total Fiber Intake (gram)	5.8 ± 2.8		5.7 ± 2.5		0.141	5.7 ± 2.8		5.7 ± 3.0		0.977	5.8 ± 2.8		5.7 ± 2.8		0.337
Total Protein Intake (gram)	58.9 ± 22.6		61.3 ± 22.6		0.02	53.7 ± 21.8		54.6 ± 22.4		0.385	56.3 ± 22.4		58.1 ± 22.8		0.015
Total Fat Intake (gram)	26.1 ± 14.5		28.4 ± 15.4		0.001	22.3 ± 13.2		22.7 ± 14.0		0.546	24.2 ± 14.0		25.6 ± 15.0		0.002

Data are presented as mean ± standard deviation or numbers (percentages, %). Abbreviation: BMI, body mass index. ^1^ Chi-square test for categorical variables and *t*-test for continuous variables were performed to examine differences between subjects with and without diabetes.

**Table 2:** The significant association results of SNPs in the *CDKAL1* gene with diabetes, fasting blood glucose, and HbA1c in Korean adults.

No.	SNP	Minor Allele	MAF		Function	Diabetes		Fasting Blood Glucose		HbA1c	
						(Controls 1484; Cases 2504)					
			Cases	Controls		OR (95% CI)	Add *p*-Value	Beta ± se	Add *p*-Value	Beta ± se	Add *p*-Value
1	rs7756992	A	0.40	0.48	Intron	0.73 (0.66–0.80)	**3.16 × 10^−10^**	−1.20 ± 0.17	**7.93 × 10^−13^**	−0.04 ± 0.01	**8.13 × 10^−5^**
2	rs9368222	A	0.48	0.44	Intron	1.35 (1.22–1.49)	**5.40 × 10^−9^**	1.25 ± 0.17	**1.18 × 10^−13^**	0.04 ± 0.01	**2.67 × 10^−6^**
3	rs2206734	T	0.48	0.44	Intron	1.35 (1.22–1.49)	**4.91 × 10^−9^**	1.30 ± 0.17	**8.51 × 10^−15^**	0.05 ± 0.01	**1.61 × 10^−7^**
4	rs9465871	T	0.40	0.48	Intron	0.73 (0.66–0.80)	**5.46 × 10^−10^**	−1.20 ± 0.17	**8.09 × 10^−13^**	−0.03 ± 0.01	**2.00 × 10^−4^**
5	rs7747752	G	0.42	0.50	Intron	0.74 (0.67–0.81)	**1.45 × 10^−9^**	−1.23 ± 0.17	**1.92 × 10^−13^**	−0.04 ± 0.01	**9.32 × 10^−5^**
6	rs9356744	C	0.48	0.44	Intron	1.35 (1.22–1.49)	**5.74 × 10^−9^**	1.25 ± 0.17	**9.29 × 10^−14^**	0.05 ± 0.01	**7.89 × 10^−7^**
7	rs6908425	T	0.16	0.21	Intron	0.68 (0.60–0.78)	**8.14 × 10^−9^**	−0.91 ± 0.22	**2.16 × 10^−5^**	−0.01 ± 0.01	0.227

Abbreviations: HbA1c, glycated hemoglobin; beta, regression coefficient; 95% CI, confidence interval; MAF, minor allele frequency; OR, odds ratio; se, standard error; SNP, single nucleotide polymorphism; Controls (no diabetes) had fasting blood glucose <110 mg/dL, and glycated hemoglobin <5.8% and were over 60 years old; Cases (diabetes) had fasting blood glucose ≥126 mg/dL or glycated hemoglobin ≥6.5% or history of diabetes diagnosis. Statistically significant values (*p*-value < 0.05) are indicated in bold and underlined.

**Table 3:** SNP in the *CDKAL1* gene and risk of diabetes by tertile of dietary protein (% energy) in Korean adults.

	Men			Women			Total		
	Protein (% Energy)			Protein (% Energy)			Protein (% Energy)		
	Tertile 1	Tertile 2	Tertile 3	Tertile 1	Tertile 2	Tertile 3	Tertile 1	Tertile 2	Tertile 3
Median	11.10	13.13	15.55	10.87	12.90	15.59	10.97	13.02	15.56
Ranges	7.76–12.15	12.15–14.09	14.09–30.05	7.47–11.86	11.87–14.01	14.01–29.14	7.47–11.99	11.99–14.07	14.07–30.05
rs7756992									
AA	1.00 (reference)	1.00 (reference)	1.00 (reference)	1.00 (reference)	1.00 (reference)	1.00 (reference)	1.00 (reference)	1.00 (reference)	1.00 (reference)
AG	1.33 (0.78–2.27)	1.53 (0.85–2.78)	0.97 (0.56–1.67)	1.40 (0.81–2.42)	0.89 (0.49–1.60)	2.08 (1.12–3.88)	1.39 (0.95–2.03)	1.17 (0.78–1.75)	1.35 (0.90–2.01)
GG	2.04 (1.16–3.60)	1.91 (1.02–3.58)	1.01 (0.55–1.87)	1.88 (1.01–3.50)	1.17 (0.64–2.14)	2.29 (1.16–4.53)	2.05 (1.35–3.09)	1.43 (0.93–2.20)	1.47 (0.94–2.30)
*p* for trend ^1^	0.034	0.674	0.937	0.278	0.475	0.011	0.001	0.113	0.842

^1^*p* for trend was obtained by using the median approach, calculating each tertile’s median as a continuous variable. Data are presented as adjusted odds ratios and 95% confidence intervals. Total models were adjusted for age, gender, BMI, waist circumference, physical activity, smoking status, drinking habits, and examination site. Men and women models were adjusted for age, BMI, waist circumference, physical activity, smoking status, drinking habits, and examination site.

**Table 4:** SNP in the *CDKAL1* gene and risk of diabetes by tertile of dietary fat (% energy) in Korean adults.

	Men			Women			Total		
	Fat (% Energy)			Fat (% Energy)			Fat (% Energy)		
	Tertile 1	Tertile 2	Tertile 3	Tertile 1	Tertile 2	Tertile 3	Tertile 1	Tertile 2	Tertile 3
Median	8.66	12.92	18.19	7.48	11.34	16.75	8.05	12.18	17.48
Ranges	2.56–11.03	11.03–15.16	15.17–35.23	2.37–9.49	9.49–13.67	13.67–43.51	2.37–10.14	10.15–14.39	14.40–43.51
rs7756992									
AA	1.00 (reference)	1.00 (reference)	1.00 (reference)	1.00 (reference)	1.00 (reference)	1.00 (reference)	1.00 (reference)	1.00 (reference)	1.00 (reference)
AG	1.26 (0.76–2.12)	1.33 (0.74–2.39)	1.16 (0.66–2.06)	1.19 (0.69–2.06)	1.29 (0.73–2.28)	1.67 (0.88–3.15)	1.12 (0.76–1.64)	1.37 (0.93–2.02)	1.45 (0.95–2.19)
GG	2.33 (1.34–4.05)	1.64 (0.88–3.06)	0.99 (0.52–1.87)	1.24 (0.68–2.25)	1.86 (1.00–3.44)	2.26 (1.11–4.59)	1.63 (1.08–2.45)	1.58 (1.03–2.41)	1.67 (1.05–2.66)
*p* for trend ^1^	0.009	0.129	0.603	0.576	0.089	0.191	0.009	0.044	0.672

^1^*p* for trend was obtained by using the median approach, calculating each tertile’s median as a continuous variable. Data are presented as adjusted odds ratios and 95% confidence intervals. Total models were adjusted for age, gender, BMI, waist circumference, physical activity, smoking status, drinking habits, and examination site. Men and women models were adjusted age, BMI, waist circumference, physical activity, smoking status, drinking habits, and examination site.

## Supplementary Materials

The following are available online at https://www.mdpi.com/1422-0067/21/16/5607/s1. Table S1: Logistic regression analysis for diabetes in Korean adults. Table S2: Linear regression analysis between SNPs in CDKAL1 gene and traits related to diabetes in Korean adults.

## Abbreviations

SNP	Single Nucleotide Polymorphism
CDKAL1	Cyclin-Dependent Kinase 5 Regulatory Subunit-Associated Protein 1-Like 1
HEXA Study	Health Examinees Study
GWAS	Genome-Wide Association Study
KoGES	Korean Genome and Epidemiology Study
BMI	Body Mass Index
HOMA-*β*	The Homeostasis Model Assessment of β-Cell Function
BDNF	Brain-Derived Neurotrophic Factor
TCF7L2	Transcription Factor 7-Like 2
HDL-Cholesterol	High Density Lipoprotein-Cholesterol
TG	Triglyceride
HbA1c	Glycated Hemoglobin
OR	Odds Ratio
AOR	Adjusted Odds Ratio
CI	Confidence Interval
MAF	Minor Allele Frequency
SE	Standard Error
HR	Hazard Ratio
PLIN	Perilipin
KDA	Korean Diabetes Association
FFQ	Food Frequency Questionnaire
HWE	Hardy-Weinberg Equilibrium
IRB	Institutional Review Board
