# Supplementary material for: Dietary Protein and Fat Intake Affects Diabetes Risk with CDKAL1 Genetic Variants in Korean Adults
Source: Int J Mol Sci. 2020 Aug 5;21(16):5607. doi: 10.3390/ijms21165607 (PMC7460637; doi:10.3390/ijms21165607)
Supplement: Supplementary file 1 [file ijms-21-05607-s001.pdf]

Table S1. Logistic regression analysis for diabetes in Korean adults[1)]

	Co–dominant		Dominant		Recessive	
	AOR (95% CI)	p	AOR (95% CI)	p	AOR (95% CI)	p
rs7756992	0.73 (0.66–0.80)	**3.16×10^{-10}**	0.66 (0.57–0.78)	**1.92×10^{-7}**	0.63 (0.53–0.75)	**2.95×10^{-7}**
rs9368222	1.35 (1.22–1.49)	**5.40×10^{-9}**	1.46 (1.24–1.71)	**2.98×10^{-6}**	1.53 (1.29–1.82)	**8.99×10^{-7}**
rs2206734	1.35 (1.22–1.49)	**4.91×10^{-9}**	1.47 (1.26–1.73)	**1.38×10^{-6}**	1.51 (1.28–1.79)	**1.69×10^{-6}**
rs9465871	0.73 (0.66–0.80)	**5.46×10^{-10}**	0.67 (0.57–0.78)	**2.51×10^{-7}**	0.63 (0.53–0.76)	**5.20×10^{-7}**
rs7747752	0.74 (0.67–0.81)	**1.45×10^{-9}**	0.66 (0.56–0.77)	**2.47×10^{-7}**	0.66 (0.56–0.79)	**2.00×10^{-6}**

[1)] Models were adjusted for age, gender, and examination site.

AOR: adjusted odds ratio; 95% CI: 95% confidence intervals.

Statistically significant values ($p < 0.05$) are indicated in bold and underlined.

Table S2. Linear regression analysis between SNPs in *CDKAL1* gene and traits related to diabetes in Korean adults[1)]

	C/C	C/R	R/R	Co–dominant	Dominant	Recessive
				p	*p*	*p*
rs7756992						
FBG	112.75±36.63	116.29±37.66	118.90±38.02	**1.91×10^{-13}**	**3.05×10^{-14}**	**2.36×10^{-5}**
HbA1c	6.12±1.21	6.15±1.14	6.26±1.14	**7.53×10^{-5}**	**1.30×10^{-5}**	**0.04**
rs9368222						
FBG	113.69±36.36	116.41±37.85	119.51±38.34	**1.21×10^{-14}**	**7.15×10^{-6}**	**1.11×10^{-16}**
HbA1c	6.10±1.14	6.16±1.15	6.31±1.17	**1.25×10^{-6}**	**8.06×10^{-3}**	**6.87×10^{-8}**
rs2206734						
FBG	113.54±36.29	116.42±37.77	119.66±38.55	**8.60×10^{-16}**	**5.40×10^{-6}**	**1.55×10^{-18}**
HbA1c	6.09±1.14	6.16±1.15	6.31±1.18	**5.88×10^{-8}**	**2.32×10^{-3}**	**2.81×10^{-9}**
rs9465871						
FBG	113.00±36.92	116.12±37.54	118.91±38.02	**2.60×10^{-13}**	**3.04×10^{-14}**	**3.88×10^{-5}**
HbA1c	6.13±1.22	6.14±1.13	6.26±1.13	**8.26×10^{-4}**	**3.24×10^{-5}**	0.07
rs7747752						
FBG	112.67±36.18	116.24±37.47	119.43±38.63	**7.07×10^{-14}**	**1.70×10^{-15}**	**3.32×10^{-5}**
HbA1c	6.12±1.19	6.15±1.14	6.27±1.14	**7.13×10^{-5}**	**6.75×10^{-6}**	0.06

Abbreviations: C, common allele; R, risk allele; FBG, fasting blood glucose; HbA1b, glycated hemoglobin. Statistically significant values (p<0.05) are indicated in bold and underlined.

[1)] Models were adjusted for age, gender, and examination site.
